# Antiplatelets versus Anticoagulants for the Treatment of Cervical Artery Dissection: Bayesian Meta-Analysis

**DOI:** 10.1371/journal.pone.0072697

**Published:** 2013-09-05

**Authors:** Hakan Sarikaya, Bruno R. da Costa, Ralf W. Baumgartner, Kathleen Duclos, Emmanuel Touzé, Jean M. de Bray, Antti Metso, Tiina Metso, Marcel Arnold, Antonio Arauz, Marcel Zwahlen, Peter Jüni

**Affiliations:** 1 Department of Neurology, University Hospital of Zurich, Zurich, Switzerland; 2 Institute of Social and Preventive Medicine, University of Bern, Bern, Switzerland; 3 CTU Bern, Bern University Hospital, Bern, Switzerland; 4 Department of Neurology, Paris Descartes University, INSERM UMR S894, and Hôpital Sainte-Anne, Paris, France; 5 Department of Neurology, University Hospital Angers, Angers, France; 6 Department of Neurology, Helsinki University Central Hospital, Helsinki, Finland; 7 Department of Neurology, University Hospital of Bern, Bern, Switzerland; 8 Stroke Clinic, Instituto Nacional de Neurología y Neurocirugía Manuel Velasco Suarez, Mexico City, Mexico; University of Regensburg, Germany

## Abstract

**Objective:**

To compare the effects of antiplatelets and anticoagulants on stroke and death in patients with acute cervical artery dissection.

**Design:**

Systematic review with Bayesian meta-analysis.

**Data Sources:**

The reviewers searched MEDLINE and EMBASE from inception to November 2012, checked reference lists, and contacted authors.

**Study Selection:**

Studies were eligible if they were randomised, quasi-randomised or observational comparisons of antiplatelets and anticoagulants in patients with cervical artery dissection.

**Data Extraction:**

Data were extracted by one reviewer and checked by another. Bayesian techniques were used to appropriately account for studies with scarce event data and imbalances in the size of comparison groups.

**Data Synthesis:**

Thirty-seven studies (1991 patients) were included. We found no randomised trial. The primary analysis revealed a large treatment effect in favour of antiplatelets for preventing the primary composite outcome of ischaemic stroke, intracranial haemorrhage or death within the first 3 months after treatment initiation (relative risk 0.32, 95% credibility interval 0.12 to 0.63), while the degree of between-study heterogeneity was moderate (τ^2^ = 0.18). In an analysis restricted to studies of higher methodological quality, the possible advantage of antiplatelets over anticoagulants was less obvious than in the main analysis (relative risk 0.73, 95% credibility interval 0.17 to 2.30).

**Conclusion:**

In view of these results and the safety advantages, easier usage and lower cost of antiplatelets, we conclude that antiplatelets should be given precedence over anticoagulants as a first line treatment in patients with cervical artery dissection unless results of an adequately powered randomised trial suggest the opposite.

## Introduction

Dissections of cervical carotid or vertebral arteries are among the most frequent causes of ischaemic stroke in young adults according to hospital-based series [1]–[3]. More than a quarter of patients with stroke caused by cervical artery dissection develop relevant disability, while almost half report a decreased quality of life [4]. The socio-economic consequences are significant, because patients with cervical artery dissection are on average 45 years of age and play an important role in private, business and social life [5]. Brain imaging studies and detection of micro-embolic signals by transcranial ultrasound in patients with cervical artery dissection suggest that arterial embolism is the main mechanism of stroke [6], [7]. Most physicians prescribe anticoagulants for stroke prevention in patients with acute cervical artery dissection, although no randomised trial has compared the safety and efficacy of anticoagulants with antiplatelets or placebo. In addition, the International Stroke Trial has shown that the potential benefit of anticoagulants is offset by an increased risk of intracranial haemorrhage in patients with acute ischaemic stroke [8].

Hitherto, three meta-analyses comparing antiplatets and anticoagulants in patients with cervical artery dissection were published [9]–[11]. All three used frequentist methods for statistical analysis. Summary estimates, uncertainty, and statistical significance vary depending on the analytical approach used. In the presence of many studies with scarce or zero events in either or both groups and imbalances in the size of comparison groups, the statistical analysis becomes challenging [12], [13]. This is the case for many of the studies comparing antiplatelets and anticoagulants in patients with cervical artery dissection. The original Cochrane Review and its recent update excluded studies with zero events in both groups [9], [11]. This approach may have biased results in either direction, particularly in view of the considerable imbalances in group sizes. Menon and colleagues included studies with zero events in both groups [10], but the analytical technique used gave undue weight to studies with zero events in both groups and is therefore likely to have biased results towards underestimating potential differences in the effects of antiplatelets and anticoagulants. Therefore, we conducted a systematic review and meta-analysis using appropriate Bayesian techniques to account for studies with scarce event data. We compared the effects of antiplatelets and anticoagulants on the composite of ischaemic stroke, intracranial haemorrhage or death as primary outcome, and determined whether estimated treatment effects differed according to the site of dissection or methodological quality of included studies.

## Methods

### Data Sources and Searches

We searched MEDLINE and EMBASE (from inception to November 2012) using a combination of keywords, text words, and specific database terms related to carotid and vertebral artery dissection and to interventions (see Appendix S1). Search strategies were developed by an experienced medical librarian in collaboration with neurologists experienced in the field of interest (HS, RWB). We used similar strategies to identify previously published systematic reviews and meta-analysis, searched clinical trial registries, screened reference lists of all retrieved reports and contacted experts in the field. There were no restrictions regarding language or publication status. See Appendix S2 for the review protocol.

### Study Selection

We included any randomised, quasi-randomised or observational study that allowed a within-study comparison of antiplatelets and anticoagulants administered for an intended duration of at least 3 months in patients with cervical artery dissection. Patients with intended shorter durations of treatment or with treatment regimens including a switch from one treatment to the other before 3 months were excluded, as were patients who underwent a surgical intervention of the dissected artery, patients with traumatic or isolated intracranial dissections, and children. Therefore, patient numbers reported here will not necessarily correspond to those previously published. Dissections were considered to be of traumatic origin in the presence of severe blunt head or neck traumas, occuring most often due to motor vehicle accidents [14], [15]. Conversely, dissections associated with minor trauma (e.g. sneezing, coughing, vomiting, minor injuries after sport or recreational activities) were considered spontaneous and included [16], [17]. Studies which did not provide 3 month follow-up data were excluded, as were case series in patients only treated with one of the interventions. Eligibility of all reports was determined by one reviewer (HS) and independently checked by one out of three other reviewers (RWB, BdC, or PJ).

### Outcome Measures

The primary outcome was the composite of ischaemic stroke, symptomatic intracranial haemorrhage or death occurring up to three months after initiation of antithrombotic treatment. Secondary outcomes were the composite of ischaemic stroke or symptomatic intracranial haemorrhage; ischaemic stroke; symptomatic intracranial haemorrhage; transient ischaemic attack; death; and the composite of ischaemic stroke or transient ischaemic attack. If three-month follow-up data were not available in published reports, we requested these data from authors.

### Data Extraction and Quality Assessment

Data on clinical outcomes and methodological quality were extracted by one reviewer (HS) and checked by another (BdC). We contacted the corresponding authors if additional information on outcome data was required for the specified follow-up period, or for the eligible patient population, overall or stratified by site of dissection (carotid or vertebral). The following components of methodological quality that may be associated with bias in therapeutic research were assessed: prospective design; enrolment of consecutive patients with cervical artery dissection; blinding of investigators responsible for the adjudication of clinical events; and inclusion of all enrolled patients in the analysis (in analogy to the intention-to-treat principle used in randomised trials). In addition, we classified studies according to their size and according to balance in the size of treatment groups. Studies were considered have balanced sizes of treatment groups if the difference in the number of patients between groups was less than fourfold. For example, a study had included 83 patients receiving aspirin and 47 receiving anticoagulants and was considered to be balanced [18]. Conversely, another study had included 9 patients receiving aspirin and 113 receiving anticoagulants and was deemed to have unbalanced sizes of treatment groups [19]. Studies were considered large if they had included more than 15 patients in each treatment group, and more than 50 patients overall. Disagreements were resolved by consensus.

### Statistical Analysis

We used a Bayesian method developed for random effects meta-analysis on the relative risk scale [20], [21]. The model adequately accounts for situations with sparse event data, including zero cells in one or both treatment groups. Monte-Carlo Markov Chain simulation methods were used to obtain posterior distributions of the relative risks (RRs) of outcomes of interest and of τ^2^. Pooled RRs were estimated from the median of the respective posterior distributions [21]. An RR below one indicates a benefit of antiplatelets as compared with anticoagulants. 95% credibility intervals (95% CrI) were obtained from the 2.5^th^ and the 97.5^th^ percentile of the posterior distribution, which can be interpreted similarly to a conventional 95% confidence interval. Between-study heterogeneity was considered low if the median of the posterior distribution of τ^2^ was 0.04 or less; τ^2^ estimates of 0.14 may be interpreted as a moderate and 0.40 as a high degree of heterogeneity between studies [22], [23]. Analyses were performed overall in all patients, and stratified according to site of dissection (carotid or vertebral). For three outcomes, we observed high degrees of heterogeneity; we identified the study contributing most to between-study heterogeneity and repeated the analyses after exclusion of this study.

For the primary composite outcome, we performed analyses of the overall population stratified by the following pre-specified methodological criteria: prospective design; enrolment of consecutive patients; inclusion of all enrolled patients in the analysis; study size; and balanced size of treatment groups. We also stratified the analysis of the primary composite outcome according to two post hoc classifications according to methodological quality: studies that satisfied all five of the above criteria (prospective design, consecutive patients, inclusion of all patients in the analysis, balanced group size and large sample size overall) versus studies that did not; studies, for which we were able to reconfirm with authors that outcome data included in our analysis were complete, versus studies for which this was not the case. All stratified analyses were accompanied by tests for interaction between study characteristic and treatment effect. Then, we performed a post hoc analysis of all outcomes restricted to studies that satisfied all five pre-specified methodological criteria [24]. After the publication of the International Stroke Trial in 1997, patients with severe stroke were more likely to receive antiplatelets than anticoagulants due to the lower risk of intracranial haemorrhage [8]. We therefore determined whether there was evidence for confounding by indication by stratifying the analysis of overall mortality according to time-point of death (≤7 days versus >7 days after symptoms onset). Finally, we re-analysed the two previous meta-analyses [10], [11] using our Bayesian random-effects model. Then, we compared results from intersecting studies, which were included in our meta-analysis as well as in those previously published, with results from studies only included in our meta-analysis, and studies only included in the previously published meta-analyses, but excluded from ours. See Appendix S3 for an extended description of statistical methods. Analyses were done using Stata version 11.0 and WinBUGS version 1.4.3 [25], [26].

## Results

**Figure 1:**
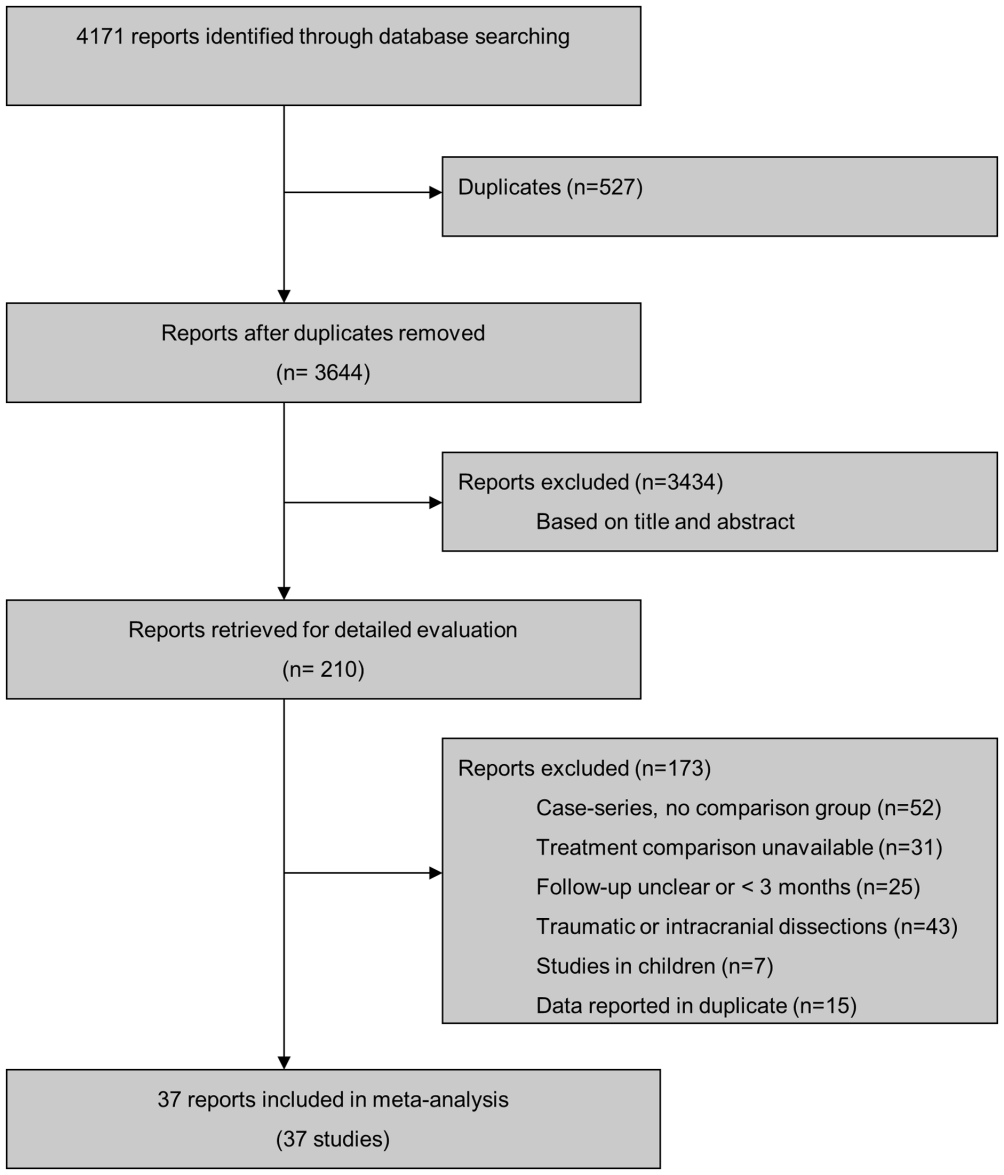
Study flow diagram.

We identified 4171 unique references through our literature search and considered 210 for detailed evaluation (Figure 1). Thirty-seven studies performed in 1991 patients fulfilled our eligibility criteria and were included. All were published as full-text articles. The median year of publication was 1998 (range, 1978 to 2012). All 37 studies were observational, allowing a comparison of antiplatelets with anticoagulants [18], [19], [27]–[61]. No randomised trial was identified. The median number of patients per study was 21 (range, 2 to 315), with 527 (26%) patients receiving antiplatelets and 1464 (74%) receiving anticoagulants. Twenty-four studies reported outcomes for 1039 patients with internal carotid artery dissection [18], [27]–[33], [35], [37]–[40], [45], [47], [49]–[51], [54]–[58], [60]. Fifteen studies reported outcomes for 532 patients with vertebral artery dissection [18], [34], [36], [42]–[44], [46], [49]–[52], [54], [55], [58], [59]. Two studies (223 patients) had included patients with internal carotid or vertebral dissection, but clinical outcomes stratified according to site of dissection were unavailable [19], [48]. Table 1 presents the methodological characteristics of included studies. Prospective design was reported in 10 studies (27%) including 874 patients [18], [44], [45], [48], [50], [52], [53], [57], [59], [61], and recruitment of consecutive patients in 19 studies (51%) with 1673 patients [18], [19], [31], [32], [34], [44]–[50], [52]–[55], [57]–[59]. Nineteen studies (51%) with a total of 1029 patients included all eligible patients in the analysis [18], [19], [27], [29], [30], [37], [38], [40]–[42], [45], [47], [48], [50], [53], [56], [57], [59], [60]. Twenty-nine studies (78%) including 1125 patients satisfied our criteria of balanced group sizes [18], [27]–[31], [33], [35]–[48], [50], [52]–[57], [61], and eight studies including 1146 patients were considered large [18], [40], [48], [49], [52], [53], [57], [61]. Four studies (11%) in 631 patients satisfied all 5 criteria and were considered to be of higher methodological quality overall [18], [48], [53], [57]. None of the included studies reported blinding of investigators responsible for the adjudication of clinical events. For 16 studies, we were able to reconfirm with investigators that outcome data included in our analysis were complete [18], [19], [37], [44], [47], [49]–[55], [57]–[60].

**Table 1:** Characteristics of identified studies.

Study, Year (Reference)	Study Population	Prospective	Consecutive	No exclusions*	Balanced group size	Large
Fisher et al. 1978 [27]	carotid	no	unclear	yes	yes	no
Luken et al. 1979 [28]	carotid	unclear	unclear	no	yes	no
Sellier et al. 1983 [29]	carotid	unclear	unclear	yes	yes	no
Vanneste et al. 1984 [30]	carotid	no	no	yes	yes	no
Mokri et al. 1986 [31]	carotid	no	yes	no	yes	no
Bogousslavsky et al. 1987 [32]	carotid	unclear	yes	no	no	no
Marx et al. 1987 [33]	carotid	unclear	unclear	no	yes	no
Mas et al. 1987 [34]	vertebral	unclear	yes	no	no	no
Landre et al. 1987 [35]	carotid	no	no	no	yes	no
Mokri et al. 1988 [36]	vertebral	unclear	unclear	no	yes	no
De Bray et al. 1989 [37]	carotid	unclear	unclear	yes	yes	no
Eljamel et al. 1990 [38]	carotid	unclear	unclear	yes	yes	no
Schievink et al. 1990 [39]	both	unclear	unclear	no	yes	no
Ast et al. 1993 [40]	carotid	no	unclear	yes	yes	yes
Landini et al. 1996 [41]	both	unclear	unclear	yes	yes	no
Pego et al. 1996 [42]	vertebral	no	unclear	yes	yes	no
Plaza et al. 1996 [43]	vertebral	unclear	unclear	no	yes	no
De Bray et al. 1997 [44]	vertebral	yes	yes	no	yes	no
Biousse et al. 1998 [45]	carotid	yes	yes	yes	yes	no
Han et al. 1998 [46]	vertebral	no	yes	no	yes	no
Engelter et al. 2000 [47]	carotid	no	yes	yes	yes	no
Beletsky et al. 2003 [48]	both	yes	yes	yes	yes	yes
Dziewas et al. 2003 [19]	both	no	yes	yes	no	no
Touzé et al. 2003 [49]	both	no	yes	no	no	yes
Caso et al. 2004 [51]	both	no	yes	yes	yes	no
Campos et al. 2004 [50]	both	yes	unclear	no	no	no
Arauz et al. 2006 [18]	both	yes	yes	yes	yes	yes
Arnold et al. 2006 [52]	vertebral	yes	yes	no	yes	yes
De Bray et al. 2007 [53]	both	yes	yes	yes	yes	yes
Nyberg et al. 2007 [54]	both	no	yes	no	yes	no
Simoes et al. 2007 [55]	both	no	yes	no	yes	no
Rigamonti et al. 2008 [56]	carotid	no	no	Yes	yes	no
Georgiadis et al. 2009 [57]	carotid	yes	yes	yes	yes	yes
Metso et al. 2009 [58]	both	no	yes	no	no	no
Arauz et al. 2010 [59]	vertebral	yes	yes	yes	no	no
Divjak et al. 2011 ^60^	carotid	no	no	yes	no	no
Kennedy et al. 2012 ^61^	both	no	no	yes	yes	yes

* No exclusions refers to no exclusions of eligible patients from the analysis.

**Figure 2:**
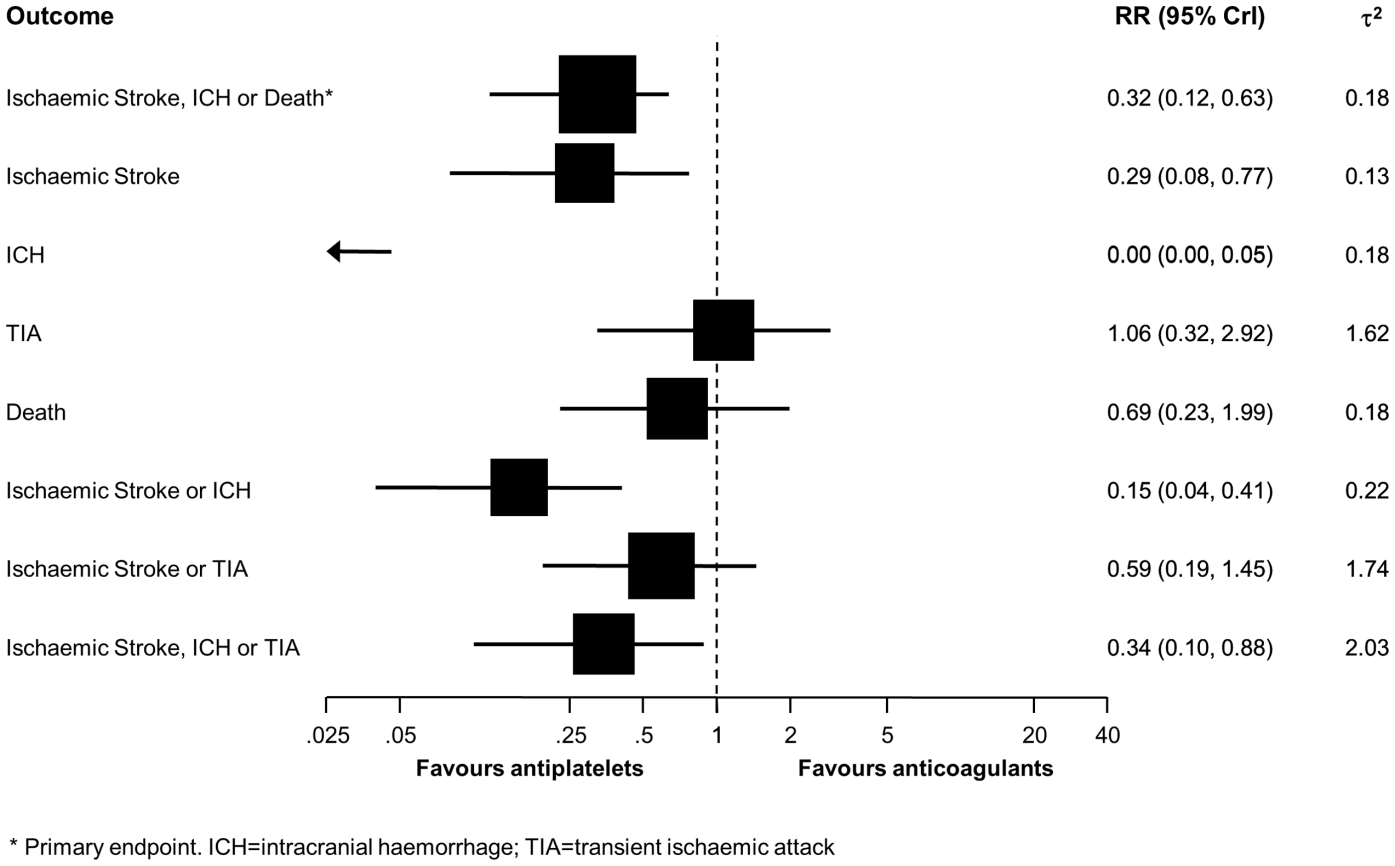
Pooled relative risks of primary and secondary outcomes comparing antiplatelets with anticoagulants. ^*^ Primary endpoint. ICH = intracranial haemorrhage; TIA = transient ischaemic attack.

Table 2 presents the clinical outcomes of all included studies. Thirty-six studies contributed to the analysis of the primary composite outcome of ischaemic stroke, intracranial haemorrhage or death [18], [19], [27]–[60]. Sixty-one patients experienced the primary composite outcome, 13 of 467 patients with antiplatelets and 48 of 1416 patients with anticoagulants. Figure 2 (top) indicates an advantage of antiplatelets over anticoagulants, with a 68% reduction in the relative risk of ischaemic stroke, intracranial haemorrhage or death afforded by antiplatelets (RR 0.32, 95% CrI 0.12 to 0.63). A τ^2^ of 0.18 indicated a moderate degree of between-study heterogeneity.

**Table 2:** Number of included patients and events reported in included studies.

	Antiplatelets									Anticoagulants								
Study, Year (Ref)	Number included	IS, ICH or death*	IS	ICH	TIA	Death	IS or ICH	IS or TIA	IS, ICH or TIA	Number included	IS, ICH or death*	IS	ICH	TIA	Death	IS or ICH	IS or TIA	IS, ICH or TIA
Fisher 1978 ^27^	1	0	0	0	0	0	0	0	0	4	2	0	1	2	1	1	2	3
Luken 1979 ^28^	1	0	0	0	1	0	0	1	1	1	0	0	0	0	0	0	0	0
Sellier 1983 ^29^	13	0	0	0	0	0	0	0	0	16	0	0	0	0	0	0	0	0
Vanneste 1984 ^30^	1	0	0	0	0	0	0	0	0	3	1	1	0	0	0	1	1	1
Mokri 1986 ^31^	9	0	0	0	0	0	0	0	0	6	0	0	0	1	0	0	1	1
Bogousslavsky 1987 ^32^	2	0	0	0	0	0	0	0	0	19	0	0	0	0	0	0	0	0
Marx 1987 ^33^	1	0	0	0	0	0	0	0	0	3	1	1	0	0	0	1	1	1
Mas 1987 ^34^	2	0	0	0	0	0	0	0	0	9	0	0	0	0	0	0	0	0
Landre E 1987 ^35^	3	1	0	0	0	1	0	0	0	1	1	0	1	0	0	1	0	1
Mokri B 1988 ^36^	9	0	0	0	0	0	0	0	0	7	0	0	0	0	0	0	0	0
De Bray 1989 ^37^	4	0	0	0	0	0	0	0	0	14	0	0	0	0	0	0	0	0
Eljamel 1990 ^38^	4	0	0	0	3	0	0	3	3	4	0	0	0	2	0	0	2	2
Schievink 1990 ^39^	5	0	0	0	0	0	0	0	0	2	0	0	0	0	0	0	0	0
Ast 1993 ^40^	21	0	0	0	0	0	0	0	0	30	1	1	0	1	0	1	2	2
Landini 1996 ^41^	5	0	0	0	0	0	0	0	0	2	0	0	0	0	0	0	0	0
Pego 1996 ^42^	2	0	0	0	0	0	0	0	0	6	0	0	0	0	0	0	0	0
Plaza 1996 ^43^	4	0	0	0	0	0	0	0	0	2	1	0	1	0	0	1	0	1
De Bray 1997 ^44^	2	0	0	0	0	0	0	0	0	2	0	0	0	0	0	0	0	0
Biousse 1998 ^45^	1	0	0	0	0	0	0	0	0	3	0	0	0	1	0	0	1	1
Han 1998 ^46^	1	0	0	0	0	0	0	0	0	1	0	0	0	0	0	0	0	0
Engelter 2000 ^47^	8	0	0	0	0	0	0	0	0	25	3	2	0	1	1	2	3	3
Beletsky 2003 ^48^	23	1	1	0	1	0	1	2	2	78	4	2	0	1	2	2	3	3
Dziewas 2003 ^19^	9	0	0	0	6	0	0	6	6	113	1	0	1	1	0	1	1	2
Touzé 2003 ^49^	18	0	0	0	0	0	0	0	0	297	4	1	0	1	3	1	2	2
Caso 2004 ^50^	20	2	1	0	0	1	1	1	1	18	2	1	0	0	1	1	1	1
Campos 2004 ^51^	6	0	0	0	0	0	0	0	0	33	4	2	0	0	2	2	2	2
Arauz 2006 ^18^	82	7	3	0	.	4	3	.	.	48	3	3	0	.	0	3	.	.
Arnold 2006 ^52^	38	2	0	0	2	2	0	2	2	24	4	3	1	3	0	4	6	7
De Bray 2007 ^53^	31	0	0	0	0	0	0	0	0	71	1	0	0	0	1	0	0	0
Nyberg 2007 ^54^	15	0	0	0	0	0	0	0	0	21	3	1	0	0	2	1	1	1
Simoes 2007 ^55^	13	0	0	0	0	0	0	0	0	29	0	0	0	0	0	0	0	0
Rigamonti 2008 ^56^	1	0	0	0	0	0	0	0	0	1	0	0	0	0	0	0	0	0
Georgiadis 2009 ^57^	96	0	0	0	2	0	0	2	2	202	3	1	2	8	0	3	9	11
Metso 2009 ^58^	8	0	0	0	0	0	0	0	0	281	9	4	3	7	2	7	11	14
Arauz 2010 ^59^	8	0	0	0	0	0	0	0	0	40	0	0	0	0	0	0	0	0
Divjak 2011 ^60^	1	0	0	0	0	0	0	0	0	20	0	0	0	0	0	0	0	0
Kennedy 2012 ^61^	59	.	1	.	3	0	.	4	.	28	.	1	.	0	0	.	1	.

* Primary endpoint.

IS = Ischaemic Stroke; ICH = intracranial haemorrhage; TIA = transient ischaemic attack.

Ref = references.

Thirty-seven studies in 527 patients with antiplatelets and 1464 patients with anticoagulants contributed to the analysis of the ischaemic stroke and death (Table 2) [18], [19], [27]–[61]. Six patients with antiplatelets and 24 patients with anticoagulants experienced an ischaemic stroke. The pooled relative risk was 0.29 (95% CrI 0.08 to 0.77) and a τ^2^ of 0.13 indicated a moderate heterogeneity between studies (Figure 2). Eight patients with antiplatelets and 15 patients with anticoagulants died during the follow-up of 3 months (pooled RR 0.69, 95% CrI 0.23 to 1.99), with a moderate heterogeneity between studies (τ^2^ = 0.18, Figure 2). The pooled RR was higher for deaths occurring within 7 days of treatment initiation (RR 1.20, 95% CrI 0.30 to 4.41) than for death occurring thereafter (RR 0.19, 95% 0.01 to 152), but a test for interaction between time of death and estimated RR did not provide strong evidence for the presence of interaction (p for interaction  = 0.14).

Thirty-six studies in 468 patients with antiplatelets and 1436 patients with anticoagulants contributed to the analyses of intracranial haemorrhage and the composite of ischaemic stroke and intracranial haemorrhage [18], [19], [27]–[60]. Intracranial haemorrhages occurred in none of the patients with antiplatelets, but in 10 patients with anticoagulants. Accordingly, the pooled RR was estimated at 0.00 (95% CrI 0.00 to 0.05), and a τ^2^ of 0.18 indicated a moderate statistical heterogeneity (Figure 2). Five patients with antiplatelets and 33 patients with anticoagulants experienced the composite of ischaemic stroke or intracranial haemorrhage. The pooled RR was 0.15 (95% CrI 0.04 to 0.41) and the heterogeneity between studies moderate (τ^2^ = 0.22, Figure 2).

Thirty-six studies in 445 patients with antiplatelets and 1416 patients with anticoagulants contributed to the analysis of transient ischaemic attack and the composite of ischaemic stroke or transient ischaemic attack [19], [27]–[61]. Eighteen patients with antiplatelets and 29 patients with anticoagulants experienced a transient ischaemic attack and there was little evidence for a difference between groups (RR 1.06, 95% CrI 0.32 to 2.92), but a high degree of heterogeneity between studies (τ^2^ = 1.62, Figure 2). Twenty-one patients with antiplatelets and 50 patients with anticoagulants experienced the composite of ischaemic stroke or transient ischaemic attack. The pooled RR was 0.59 (95% CrI 0.19 to 1.45), but the heterogeneity between studies was large (τ^2^ = 1.74).

Thirty-five studies in 386 patients with antiplatelets and 1388 patients with anticoagulants contributed to the analysis of the composite outcome of ischaemic stroke, intracranial haemorrhage or transient ischaemic attack.) [19], [27]–[60]. Seventeen patients with antiplatelets and 59 patients with anticoagulants experienced the composite of ischaemic stroke, intracranial haemorrhage or transient ischaemic attack. The pooled RR was 0.34 (95% CrI 0.10 to 0.88) and a τ^2^ of 2.03 again indicated large statistical heterogeneity.

**Figure 3:**
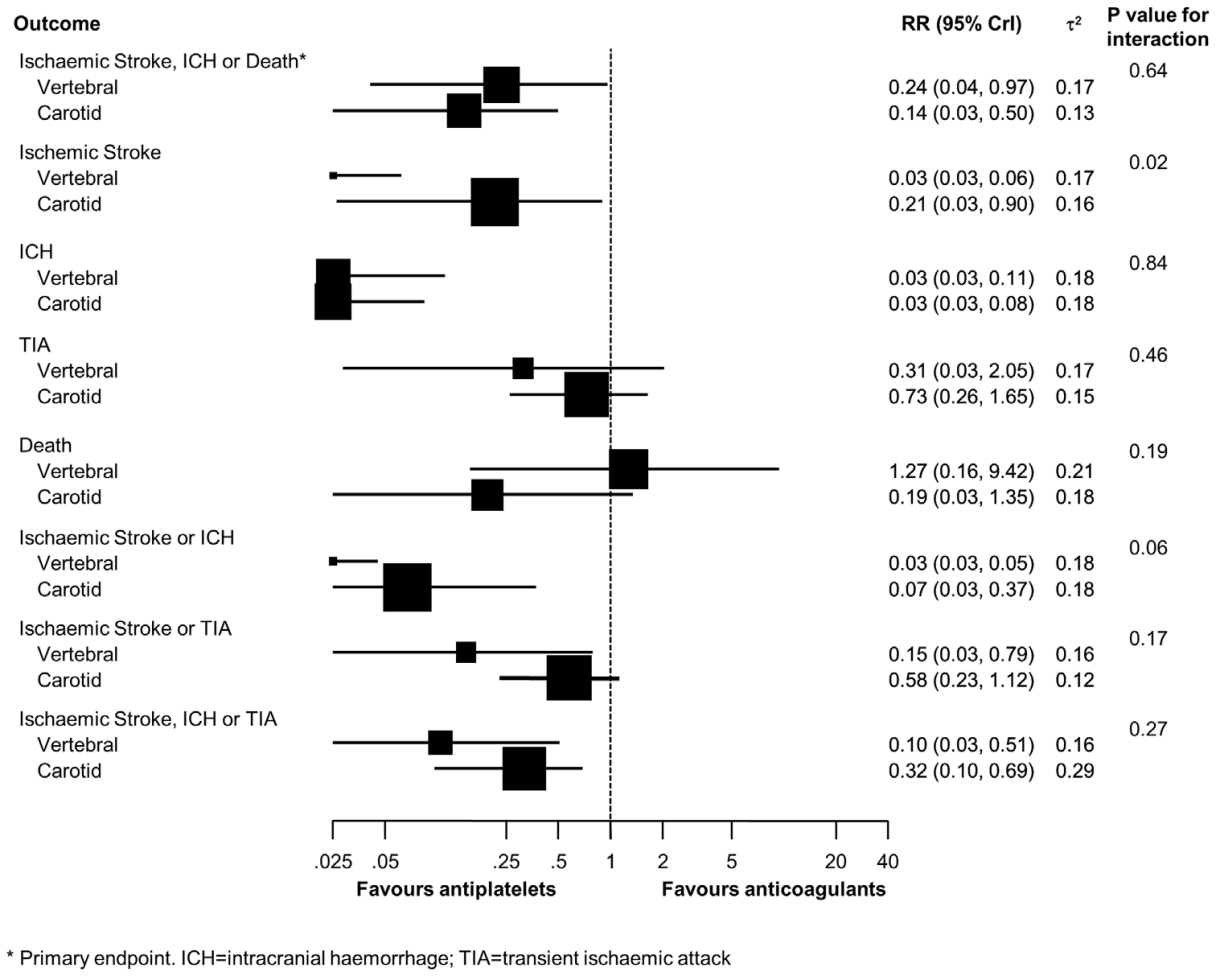
Analyses stratified according to site of dissection. * Primary endpoint. ICH = intracranial haemorrhage; TIA = transient ischaemic attack.

Figure 3 presents results of analyses stratified according to site of dissection. Estimates varied to some extent according to dissection site, but tests for interaction were negative for 7 out of the 8 outcomes, including the primary composite of ischaemic stroke, intracranial haemorrhage or death. The test for interaction between site of dissection and estimated treatment effect was positive, however, for ischaemic stroke (p for interaction  = 0.02), suggesting a more pronounced benefit of antiplatelets in patients with vertebral dissection.

Table 3 presents results from analyses of the primary outcome stratified according to study characteristics. Throughout, estimated relative risks were nearer 1 in studies that satisfied a methodological criterion. Tests for interaction between estimated relative risks and methodological criteria formally reached statistical significance for prospective study design (p for interaction  = 0.01) and balanced group size (p for interaction  = 0.04), and a statistical trend for consecutive recruitment of patients (p for interaction  = 0.06).

**Table 3:** Stratified analyses of the primary outcome according to methodological quality.

Variable	Total Studies, n	Number of patients included	RR (95% CrI)	τ^2^ *	P Value for interaction
**All studies**	37	1991	0.32 (0.12−0.63)	0.18	
**Prospective design**				0.13	0.01
Yes	10	874	0.62 (0.22−1.47)		
No	27	1117	0.05 (0.00−0.30)		
**Consecutive enrolment of patients**				0.16	0.06
Yes	19	1673	0.45 (0.16−0.98)		
No	18	318	0.09 (0.00−0.52)		
**Intention-to-treat analysis**				0.13	0.06
Yes	19	1029	0.52 (0.16−1.18)		
No	18	962	0.15 (0.03−0.51)		
**Balanced size of treatment groups**				0.15	0.04
Yes	29	1131	0.35 (0.13−0.67)		
No	8	860	0.00 (0.00−0.55)		
**Large sample size**				0.17	0.08
Yes	8	1146	0.51 (0.15−1.31)		
No	29	845	0.17 (0.04−0.51)		

* Note that only one τ^2^ was estimated per outcome.

Table 4 shows results from a post hoc analysis of all outcomes restricted to studies that satisfied all five pre-specified methodological criteria. Compared with the analysis of all studies, estimates became less beneficial for antiplatelets on all outcomes except transient ischaemic attack. 95% CrI all overlapped the null effect at a RR of 1 for all outcomes, except intracranial haemorrhage. Studies that satisfied all five pre-specified methodological criteria showed a pooled RR of 0.73 (95% CrI 0.17 to 2.30), studies that satisfied four criteria or less showed a pooled RR of 0.20 (95% CrI 0.06 to 0.47; p for interaction  = 0.07). For the 16 studies, for which we were able to reconfirm with investigators that outcome data included in our analysis were complete we found a pooled RR of 0.40 (95% CrI 0.12 to 0.91), in remaining studies a pooled RR of 0.17 (95% CrI 0.02 to 0.60; p for interaction 0.17).

**Table 4:** Pooled relative risks of primary and secondary outcomes found in studies satisfying all 5 methodological criteria.

Outcome	RR (95% CrI)	τ^2^
Ischaemic Stroke	0.65 (0.12−3.05)	0.15
ICH	0.00 (0.00−0.47)	0.18
TIA	0.73 (0.12−3.97)	0.18
Death	1.46 (0.17−10.79)	0.26
Ischaemic Stroke or ICH	0.52 (0.10−2.16)	0.16
Ischaemic Stroke or TIA	0.79 (0.17−3.31)	0.19
Ischaemic Stroke, ICH or TIA	0.68 (0.14−2.98)	0.21

* Primary endpoint.

ICH = intracranial haemorrhage; TIA = transient ischaemic attack.

Table 5 shows a comparison of results of our and previous meta-analyses [10], [11]. Engelter and Lyrer used a fixed-effect model to derive pooled Peto odds ratios. They found clinically relevant, but non-significant reductions in the odds of stroke (OR 0.63, 95% CI 0.21 to 1.86) and intracranial haemorrhage (OR 0.25, 95% CI 0.02 to 3.36), but a clinically relevant trend towards higher overall mortality associated with antiplatelets as compared with anticoagulants (OR 2.02, 95% CI 0.62 to 6.60). Our re-analysis of Engelter and Lyrer's data using our Bayesian random-effects model (Table 5, top) shows a pronounced, albeit non-significant 55% relative reduction of stroke and a pronounced, statistically significant 100% relative risk reduction of intracranial haemorrhage. The 40% relative risk increase of death was non-significant and less pronounced than reported by Engelter and Lyrer [11] .The 20 intersecting studies in 1109 patients included in their and in our meta-analysis were less beneficial for antiplatelets (RR of stroke 0.64, 95% CrI 0.18 to 2.14) than the 16 studies in 176 patients included only in their meta-analysis (RR 0.45, 95% CrI 0.15 to 1.41) and the 17 studies in 882 patients included only in our meta-analysis (RR 0.19, 95% CrI 0.02 to 0.97, Table 5, middle). Menon et al used a fixed-effect model, which was not further specified. They found a small risk difference of −0.01 for stroke (95% CI −0.06 to 0.04), which was slightly in favour of antiplatelets, and a more pronounced risk difference in opposite direction of 0.05 in favour of anticoagulants for the composite of stroke or TIA (95% CI −0.01 to 0.11). In our re-analysis of their data (Table 5, top), we also found opposite directions of effects. However, on a relative risk scale, these opposite effects had the same magnitude: a 49% relative risk reduction for stroke and a 48% relative risk increase for the composite of stroke or TIA associated with antiplatelets, with considerable imprecision of both estimates, as evidenced by wide 95% credibility intervals. The 18 intersecting studies in 553 patients included in their and our meta-analysis (RR of stroke 0.48, 95% CrI 0.10 to 1.76) and the 16 studies in 209 patients included only in their meta-analysis (RR 0.65, 95% CrI 0.02 to 13.72) were less beneficial for antiplatelets than the 19 studies in 1438 patients included only in our meta-analysis (0.15, 95% CrI 0.02 to 0.70, Table 5, bottom).

**Table 5:** Influence of in- or exclusion of studies on estimated effects.

			RR (95% CrI)			
	Number of studies	Number of patients	Ischaemic Stroke	ICH	Death	Ischaemic Stroke or TIA
Our meta-analysis, all included studies	37	1991	0.29 (0.08−0.77)	0.00 (0.00−0.05)	0.69 (0.23−1.99)	0.59 (0.19−1.45)
Engelter et al's meta-analysis, all included studies	36	1285	0.45 (0.15−1.41)	0.00 (0.00−0.36)	1.40 (0.46−3.79)	n/a
Menon et al's meta-analysis, all included studies	34	762	0.51 (0.13−1.62)	n/a	n/a	1.48 (0.41−4.33)
*Engelter et al's versus our meta-analysis*						
Studies included in Engelter et al's and our meta-analysis	20	1109	0.64 (0.18−2.14)	0.00 (0.00−0.15)	1.80 (0.38−9.15)	n/a
Studies included in Engelter et al's but not in our meta-analysis	16	176	0.45 (0.15−1.41)	0.00 (0.00−26.68)	0.96 (0.13−4.32)	n/a
Studies included in our, but not in Engelter et al's meta-analysis	17	882	0.19 (0.02−0.97)	0.00 (0.001−0.003)	0.33 (0.03−1.80)	n/a
*Menon et al's versus our meta-analysis*						
Studies included in Menon et al's and our meta-analysis	18	553	0.48 (0.10−1.76)	n/a	n/a	2.00 (0.44−7.52)
Studies included in Menon et al's but not in our meta-analysis	16	209	0.65 (0.02−13.72)	n/a	n/a	0.76 (0.10−4.05)
Studies included in our, but not in Menon et al's meta-analysis	19	1438	0.15 (0.02−0.70)	n/a	n/a	0.41 (0.14−1.03)

Outcomes included if reported in at least two out of the three available meta-analyses.

ICH = intracranial haemorrhage; TIA = transient ischaemic attack.

Sensitivity analyses using different statistical methods are reported in Appendix S4. For the primary composite endpoint of stroke, intracranial haemorrhage or death, we found point estimates and precision much the same for all five methods used. This was also the case for stroke, death, and the composite of stroke or intracranial haemorrhage. For remaining outcomes, there were some differences in point estimates or precision, but overlapping credibility intervals suggested compatibility of estimates.

## Discussion

In this meta-analysis of 37 observational studies comparing antiplatelets with anticoagulants in 1991 patients with cervical artery dissection, we found evidence to suggest a clinically relevant advantage of antiplatelets over anticoagulants on the primary outcome (composite of ischaemic stroke, intracranial haemorrhage or death), and 4 out of 7 secondary outcomes. When we stratified according to components of study quality, we found the benefit of antiplatelets considerably less pronounced in studies of higher methodological quality. Tests for interaction between estimated relative risks and study characteristics formally reached statistical significance for prospective study design and balanced group size, and showed a statistical trend for consecutive recruitment of patients and analysis in accordance with the intention-to-treat principle. In an analysis restricted to studies, which satisfied all pre-specified methodological criteria, credibility intervals were wide for all outcomes, except for intracranial haemorrhage, and were compatible with both, a substantial advantage or disadvantage of antiplatelets over anticoagulants. Furthermore, we stratified analyses according to the site of dissection, i.e. carotid or vertebral artery. This analysis showed similar results in the two groups, even though tests for interaction between the site of dissection and the estimated treatment effect were positive for ischaemic stroke and the composite of ischaemic stroke or intracranial haemorrhage. None of the numerous analyses provided robust evidence that anticoagulants are more beneficial than antiplatelets in patients with cervical artery dissection. The risk of intracranial hemorrhage was lower in the antiplatelet group and may be considered to contradict contemporary trials, which found aspirin in patients with atrial fibrillation not safer than warfarin [62], [63]. This apparent contradiction may be explained by the characteristics of included patients (age, comorbidity and severity of stroke) and timing of treatment initiation (immediate versus delayed): patients with cervical artery dissection are typically younger and have less comorbid conditions than patients with atrial fibrillation at risk of stroke considered for antiplatelet treatment, but typically experience severe strokes with large brain tissue infarction. If treatment is initiated immediately, these patients may be more likely to suffer intracranial haemorrhage with anticoagulation as compared to antiplatelet treatment [8]. This notion is in accordance with current guidelines, which recommend against full-dose anticoagulation in patients with acute ischaemic stroke [64], [65].

In our view, the major strength of this study is the use of Bayesian techniques to address the challenge of studies with scarce event data and studies with imbalanced sizes of treatment groups [21]. The major limitation is the complete lack of randomised trials comparing antiplatelets and anticoagulants in patients with cervical dissection and the variation in methodological quality of the observational studies included. We addressed this by performing analyses stratified according to methodological quality and found evidence for overestimations of the benefit of antiplatelets in studies of lower methodological quality. In an analysis restricted to studies of higher methodological quality, the possible advantage of antiplatelets over anticoagulants was less obvious than in the main analysis. Observational studies may be subject to confounding by indication: patients with extensive stroke at baseline and substantially increased risk of subsequent intracranial haemorrhage may be more likely to receive antiplatelets than anticoagulants [66], [67]. These patients typically have a poor prognosis and may die early after initiation of antithrombotic treatment. We were unable to formally compare stroke severity between patients with antiplatelet and anticoagulant treatment at baseline because of the low quality of reporting, but attempted to address this indirectly by performing an additional analysis of mortality data stratified according to time of death. During the first seven days after treatment initiation, we found a trend towards more deaths in the antiplatelet group, whereas a trend into the opposite direction was observed for the subsequent period up to 3 months. This observation indeed suggests confounding by indication, which may have introduced bias against antiplatelets, although a statistical test for interaction was negative. Conversely, patients may have been more likely to receive antiplatelets if they had lower degree stenoses of cerebral arteries or few clinical symptoms only [57]. This could have biased results in favour of antiplatelets, but again, the lack of information on baseline characteristics prevented us from addressing this formally. Results from adjusted analysis were available only for one study [57], which used the presence or absence of cerebral ischemic symptoms as covariate. The adjusted estimate for the composite of ischemic stroke, TIA or transient monocular blindness or retinal infarction showed a trend in favour of antiplatelets (OR 0.38, 95% CI 0.08 to 1.67) [57]. An alternative attempt to address comparability of groups was the classification of studies according to the balance in the size of treatment groups. Some might argue that this criterion is not obviously related to bias. We pre-specified it as a proxy for pronounced differences in treatment indication in a specific study. For example, in the study by Dziewas et al, only 7% of patients (9 out of 122) [19] had received antiplatelets as opposed to 64% in the study by Arauz et al (83 out of 130) [18]. We consider it more likely in the first than in the second study that pronounced differences in indication for treatment introduced bias: the 7% of patients who received antiplatelets are likely to be highly selected and not comparable with the remaining 93% who received anticoagulation. We found that this approach towards addressing comparability of groups was more suitable than a direct comparison of relevant patient characteristics at baseline between groups because of the limited quality of reporting of included studies. A further source of bias is the potential for selective reporting of outcomes [68] ,which may have biased results in favour of either treatment. We addressed this in a post hoc analysis, distinguishing between studies, for which we were able to reconfirm with authors that outcome data included in our analysis were complete, and studies for which this was not the case. There was little evidence for a difference in estimated effects, and studies with complete outcome data showed a robust advantage of antiplatelets.

We were also unable to address the fact that safety and efficacy of anticoagulation is associated with control of international normalized ratio since included studies did not provide the necessary information [69]. Consequently, we cannot exclude that patients with appropriate control of their international normalized ratio will fair better than patients included in our study. Another limitation may concern the exclusion of patients with a traumatic dissection, as classification into traumatic or spontaneous form may be arbitrary in some cases. Furthermore, the state of the dissected artery (stenosis vs. occlusion), which may be associated with both, the choice of antithrombotic treatment and prognosis, was not reported in most studies. Finally, the degree of heterogeneity between studies observed for three outcomes (transitory ischaemic attacks, and two composite endpoints including transitory ischaemic attacks as one of their components) was high. We identified one study to contribute most to heterogeneity [19]: it was small, had unbalanced group sizes and an unusually high rate of transitory ischaemic attacks in patients treated with antiplatelets. We performed a sensitivity analysis after exclusion of this study and found heterogeneity decreased, but emphasise the purely explorative character of this analysis along with the fact that remaining heterogeneity between studies was still moderate.

Three meta-analyses have been published so far [9]–[11]. All used frequentist methods for statistical analyses. The original Cochrane Review and its recent update used a fixed effect model to derive pooled Peto odd ratios excluding studies with zero events in both groups [9], [11], [70]. In view of the considerable imbalances in group sizes, this approach may have introduced bias in favour of either treatment. The Cochrane Review included only studies reporting on patients with cervical carotid artery dissection, whereas we included also patients with vertebral artery dissection. When analyzing the primary outcome in our study, we found similar relative risks for carotid (RR 0.14, 95% CrI 0.03 to 0.50) and vertebral dissections (RR 0.24, 95% CrI 0.04 to 0.97), which suggests that a combined analysis of the two sites of dissections is viable. Another important difference is the choice of primary outcomes, which were overall mortality and the composite of death or disability in the Cochrane Review [9], [11]. For death, they found a pooled odds ratio of 2.02 (95% confidence interval 0.62 to 6.60), whereas our pooled relative risk was 0.69 (95% CrI 0.23 to 1.99). This difference in pooled estimates may be explained by chance alone, differences in study selection or differences in the analytical approaches used. Differences in study selection mainly occurred because of more stringent selection criteria in our study, requiring 3-month follow-up data. This follow-up duration was chosen in view of the frequent change from anticoagulants to antiplatelets in routine clinical practice that typically occurs after completion of the first three months of treatment. Other reasons for differing study selection include a more up to date literature search and the discussed inclusion of both, patients with carotid and vertebral dissection, even though we deem it unlikely that differences in results between the Cochrane Review and our study can be fully explained by dissection site. Re-analyses of data included in the Cochrane Review and our meta-analysis reported in Table 5 in comparison with original results [11] suggested that the differences between their and our results could be explained by both, differences in statistical methods and differences in study identification and selection. We emphasise, however, that widely overlapping uncertainty intervals indicate that our results for stroke, intracranial haemorrhage and death are compatible with both, the Bayesian re-analysis of the data of the Cochrane Review and the original results published of the Cochrane Review. We did not analyse the composite of death or disability, since disability was typically not reported at 3 months, but it is obvious that results on this long term outcome (odds ratio of 1.77 in favour of anticoagulants, 95% CI 0.98 to 3.22) reported in Cochrane Review are in opposition to the majority of our short to midterm results. This discrepancy could be explained by the different nature of outcomes, but may also be related to challenges in interpreting longer term data of included observational studies: clinical practice frequently involves a switch from anticoagulation to antiplatelets after 3 to 6 months, thus the true association of outcomes with type of antithrombotic treatment is difficult to determine after this period.

Menon and colleagues used a fixed effect model to derive risk differences and included studies with zero events in both groups [10]. They observed no relevant difference between antiplatelets and anticoagulants. This is not surprising because about half of the studies included had null events in both groups with an estimated risk difference of zero [10]. Their analytical approach used gave undue weight to these studies and is likely to have biased results towards underestimating potential differences [12]. In addition, they confined the follow-up duration to one month, which will have led to the exclusion of clinical events and reduced statistical power as compared with our analysis. Re-analyses of data included in Menon et al's and our meta-analysis (reported in Table 5) in comparison with original results [10] suggested that the differences can be explained by differences in statistical methods, study identification and selection, even though the impact of study identification and selection was more pronounced, with a nearly threefold increase in the number of patients in our as compared with Menon et al's analysis. Studies included either in the Cochrane Review [11] or in the meta-analysis by Menon et al [10], which were excluded from our analysis typically because of short follow-up duration or unclear reporting of type of treatment or outcome that could be resolved with authors, were small and had only a small number of primary outcome events.

Currently, two small-scale randomised trials comparing anticoagulants and antiplatelets in patients with cervical artery dissection are ongoing. The Cervical Artery Dissection in Stroke Study (CADISS) aims at including 250 patients. 215 patients have been randomised by 3^rd^ December 2012 [71]. The other trial, a pilot study in 20 patients, has completed recruitment [72]. Both trials are pilot studies, not set up to formally compare antiplatelets with anticoagulants in terms of patient-relevant clinical outcomes.

Results of our overall analysis suggest an advantage of antiplatelets over anticoagulants on nearly all outcomes, which contradict the current preferential use of anticoagulants in routine clinical practice for patients with cervical artery dissection. Stratified analyses according to methodological quality showed less pronounced advantages of antiplatelets in studies of higher methodological quality, with point estimates nearer the line of no difference at 1 and credibility intervals compatible with both relevant advantages and disadvantages of antiplatelets over anticoagulants. This suggests equipoise between the two types of treatments and calls for a carefully designed, adequately powered randomised multicenter trial. A sample size of 2100 patients per group will provide 80% power at a two-sided alpha of 0.05 to detect a 30% relative risk reduction in the primary composite outcome of stroke, intracranial haemorrhage or death from 7% in patients receiving anticoagulants to 4.9% in patients receiving antiplatelets. Performing such a trial is difficult, time-consuming and costly. Cervical artery dissection is a typical example of an acute rare condition where, globally, it would be possible to recruit about 4000 patients to conduct a properly powered randomised trial within one or two years. However, given that each center would contribute only 1–2 patients per year, literally thousands of centers would be required, which makes the costs of initiation and logistics of such a trial prohibitive. Novel approaches might be needed, with an international trials consortium performing a series of trials in patients with different types of rare conditions, with standard procedures, common logistics, and simplified requirements for approval by local research ethics committees for the entire series of trials. For now, considering that discrepancies between randomised trials and observational studies may be less pronounced than theoretically expected [73], we call for carefully conducted prospective cohort studies to fill the evidence gap until randomised evidence becomes available.

Oral anticoagulants are widely used in routine clinical practice in patients with cervical artery dissection. They are about 15 times more expensive than aspirin [74], the classical antiplatelet used in clinical practice for the treatment of cervical artery dissection, require a higher degree of compliance, dependent on frequent laboratory testing and carry a higher risk of intracranial haemorrhage than antiplatelets. The pharmacokinetic profile of oral anticoagulants is variable and there are multiple interactions with drugs and food, which frequently result in poor control of the INR [75]. As compared to conventional Vitamin K antagonists, anticoagulation with the novel factor Xa or thrombin inhibitors were found more effective in stroke prevention in patients with atrial fibrillation and more practical for handling.^76,77^ The safety and effectiveness of these new anticoagulants in patients with cervical artery dissection will need to be investigated in randomised trials. Considering these practical and theoretical disadvantages of anticoagulants and our meta-analysis, which clearly provides no evidence for a superiority of anticoagulants over antiplatelets, even pointing towards a harmful effect of anticoagulants, we question the preferential use of anticoagulants as a first line treatment in patients with cervical artery dissection and conclude that antiplatelets should be given precedence instead, unless results of an adequately powered randomised trial suggest the opposite.

## Supporting Information

Appendix S1 **Literature search strategy.**
(DOCX)

Appendix S2 **Review Protocol.**
(DOCX)

Appendix S3 **Extended description of statistical methods.**
(DOCX)

Appendix S4 **Different approaches for estimating summary effect.**
(DOC)
