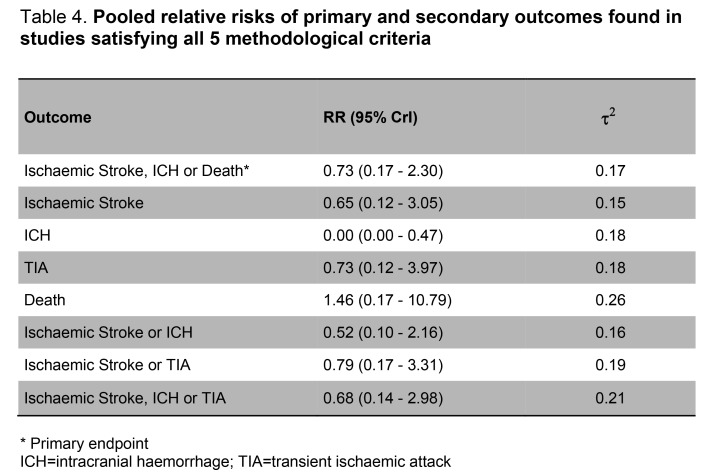# Correction: Antiplatelets versus Anticoagulants for the Treatment of Cervical Artery Dissection: Bayesian Meta-Analysis

**DOI:** 10.1371/annotation/5be41b8a-b56f-43f7-8a57-b30bd7f90d79

**Published:** 2014-01-02

**Authors:** Hakan Sarikaya, Bruno R. da Costa, Ralf W. Baumgartner, Kathleen Duclos, Emmanuel Touzé, Jean M. de Bray, Antti Metso, Tiina Metso, Marcel Arnold, Antonio Arauz, Marcel Zwahlen, Peter Jüni

A row is missing from Table 4. Please see the corrected Table 4 here: 

**Figure:**