# Supplementary material for: Antiplatelets versus Anticoagulants for the Treatment of Cervical Artery Dissection: Bayesian Meta-Analysis
Source: PLoS One. 2013 Sep 5;8(9):e72697. doi: 10.1371/journal.pone.0072697 (PMC3764185; doi:10.1371/journal.pone.0072697)
Supplement: Appendix S1 — Literature search strategy. (DOCX) [file pone.0072697.s001.docx]

S1. **Literature search strategy**

| **Medline** | |
| --- | --- |
| **Step** | **Search Strategy** |
| 1 | exp carotid artery injuries/ OR carotid artery, internal, dissection/ |
| 2 | exp Vertebral Artery Dissection/ |
| 3 | (carotid adj5 (injur* OR dissection OR trauma*)).tw. |
| 4 | (vertebral adj5 (injur* OR dissection OR trauma*)).tw. |
| 5 | OR/1-4 |
| 6 | exp carotid arteries/ |
| 7 | carotid artery diseases/ |
| 8 | carotid artery thrombosis/ |
| 9 | carotid*.tw. |
| 10 | exp Vertebral Artery / |
| 11 | vertebral*.tw. |
| 12 | OR/6-11 |
| 13 | exp aneurysm, dissecting/ OR aneurysm, false/ OR exp aneurysm, ruptured/ |
| 14 | exp wounds, nonpenetrating/ |
| 15 | (traumatic adj5 (dissection OR aneurysm OR pseudoaneurysm)).tw. |
| 16 | (blunt adj5 (injur* OR trauma)).tw. |
| 17 | dissecting aneurysm.tw. |
| 18 | rupture, spontaneous/ OR rupture/ |
| 19 | spontaneous dissection.tw. |
| 20 | OR/13-19 |
| 21 | 12 AND 20 |
| 22 | 5 OR 21 |
| 23 | exp platelet aggregation inhibitors/ |
| 24 | blood platelets/de |
| 25 | platelet aggregation/de |
| 26 | exp fibrinolytic agents/ |
| 27 | exp anticoagulants/ |
| 28 | Thrombolytic therapy/ |
| 29 | exp thromboembolism/dt |
| 30 | thrombosis/dt |
| 31 | (antiplatelet* OR antithrom* OR anticoag*).tw. |
| 32 | (aspirin OR acetylsalicylic acid OR indobufen).tw. |
| 33 | (dipyridamole OR ticlopidine OR clopidogrel OR sulfinpyrazone OR sulphinpyrazone).tw. |
| 34 | (heparin* OR coumarin* OR coumadin* OR warfarin).tw. |
| 35 | OR/23-34 |
| 36 | 35 AND 22 |
| 37 | limit 36 to humans |
|  | |
| **Embase** | |
| **Step** | **Search Strategy** |
| 1 | 'carotid artery thrombosis'/dm_dt OR 'carotid artery obstruction'/dm_dt OR 'carotid artery aneurysm'/dm_dt OR 'internal carotid artery occlusion'/dm_dt OR 'internal carotid artery aneurysm'/dm_dt OR 'vertebral artery stenosis'/dm_dt |
| 2 | 'carotid artery'/exp OR 'carotid artery disease'/exp OR 'vertebral artery'/exp |
| 3 | carotid:ab,ti OR vertebral:ab,ti |
| 4 | #2 OR #3 |
| 5 | 'artery dissection'/de OR 'artery injury'/de OR 'artery rupture'/de OR 'artery thrombosis'/de |
| 6 | 'artery'/exp AND wall AND 'dissection'/exp OR 'artery wall dissection' |
| 7 | 'blood vessel injury'/de OR 'false aneurysm'/de OR 'blunt trauma'/de OR 'rupture'/de |
| 8 | ('traumatic' NEAR/5 'dissection'):ab,ti OR ('traumatic' NEAR/5 'aneurysm'):ab,ti OR ('traumatic' NEAR/5 'pseudoaneurysm'):ab,ti OR ('blunt' NEAR/5 'injury'):ab,ti OR ('blunt' NEAR/5 'injuries'):ab,ti OR ('blunt' NEAR/5 'injured'):ab,ti OR ('blunt' NEAR/5 'trauma'):ab,ti OR 'dissecting aneurysm':ab,ti OR 'spontaneous dissection':ab,ti |
| 9 | # 5 OR # 6 OR #7 OR #8 |
| 10 | #4 AND #9 |
| 11 | ('carotid' NEAR/5 'trauma'):ab,ti OR ('carotid' NEAR/5 'traumatic'):ab,ti OR ('carotid' NEAR/5 'injury'):ab,ti OR ('carotid' NEAR/5 'injuries'):ab,ti OR ('carotid' NEAR/5 'injured'):ab,ti OR ('carotid' NEAR/5 'dissection'):ab,ti |
| 12 | ('vertebral' NEAR/5 'trauma'):ab,ti OR ('vertebral' NEAR/5 'traumatic'):ab,ti OR ('vertebral' NEAR/5 'injury'):ab,ti OR ('vertebral' NEAR/5 'injuries'):ab,ti OR ('vertebral' NEAR/5 'injured'):ab,ti |
| 13 | #1 OR #10 OR #11 OR #12 |
| 14 | 'anticoagulant agent'/exp OR 'antithrombocytic agent'/exp OR 'thrombocyte aggregation'/de OR 'thrombocyte'/de OR 'fibrinolytic therapy'/de OR 'thromboembolism'/de |
| 15 | antiplatelet*:ab,ti OR anticoagulant*:ab,ti OR antithromb*:ab,ti |
| 16 | aspirin:ab,ti OR 'acetylsalicylic acid':ab,ti OR indobufen:ab,ti |
| 17 | dipyridamole:ab,ti OR ticlopidine:ab,ti OR clopidogrel:ab,ti OR sulfinpyrazone:ab,ti OR sulphinpyrazone:ab,ti |
| 18 | heparin*:ab,ti OR coumarin*:ab,ti OR coumadin*:ab,ti OR warfarin:ab,ti |
| 19 | #14 OR # 15 OR #16 OR #17 OR #18 |
| 20 | #13 AND #19 |
| 21 | #13 AND #19 AND [humans]/lim |
