# Supplementary material for: Antiplatelets versus Anticoagulants for the Treatment of Cervical Artery Dissection: Bayesian Meta-Analysis
Source: PLoS One. 2013 Sep 5;8(9):e72697. doi: 10.1371/journal.pone.0072697 (PMC3764185; doi:10.1371/journal.pone.0072697)
Supplement: Appendix S2 — Review Protocol. (DOCX) [file pone.0072697.s002.docx]

S2*.* **Review Protocol**

Research team responsible for protocol development

- Hakan Sarikaya
- Ralph Baumgartner
- Bruno da Costa
- Peter Jüni

Literature search

- Databases: Medline and Embase
- Search period: from inception of databases to present
- Other sources: reference list of included studies, clinical trial registries, and experts in the field

Study eligibility (PICO format)

- Population: patients with cervical artery dissection
- Intervention: anticoagulants
- Comparator: antiplatelets
- Outcome: composite of ischemic stroke, symptomatic intracranial hemorrhage or death. Time point of interest: three months after initiation of antithrombotic treatment.
- Design: observational studies or randomized controlled trials

Outcomes of interest

- Primary outcome: composite of ischemic stroke, symptomatic intracranial hemorrhage or death. Time point of interest: three months after initiation of antithrombotic treatment.
- Secondary outcomes: ischaemic stroke; symptomatic intracranial haemorrhage; TIA; death; composite of ischaemic stroke or symptomatic intracranial haemorrhage; and the composite of ischaemic stroke or TIA.

Data extraction

- One reviewer will extract data from all trials. Extracted data will be checked for accuracy by an independent reviewer.
- We may contact authors if data needed for the analysis is not reported

Methodological quality

We will assess the following items:

- consecutive sampling,
- prospective design,
- blinded adjudication of clinical events,
- balance of group size,
- study sample size (i.e. small and large studies),
- intention-to-treat analysis

Statistical analysis

- Pooling of risk ratios using Bayesian random-effects meta-analysis
- Tau-squared will be used to quantify between-study heterogeneity. Cut-offs for the interpretation of tau-squared: 0.04 (low), 0.14 (moderate), 0.40 (large)
- Stratified analyses with test for interaction will be performed according to: site of dissection (i.e. carotid or vertebral), time-point of death (≤7 days Vs >7 days after symptoms onset), and methodological characteristics
