# Supplementary material for: Antiplatelets versus Anticoagulants for the Treatment of Cervical Artery Dissection: Bayesian Meta-Analysis
Source: PLoS One. 2013 Sep 5;8(9):e72697. doi: 10.1371/journal.pone.0072697 (PMC3764185; doi:10.1371/journal.pone.0072697)
Supplement: Appendix S3 — Extended description of statistical methods. (DOCX) [file pone.0072697.s003.docx]

S3*.* **Extended description of statistical methods**

We used a Bayesian method developed for random effects meta-analysis on the log odds ratio scale,[^1^](#_ENREF_1) which was extended for log risk ratios.[^2^](#_ENREF_2)

For each study i, δ_i_ quantifies the shift on the log of the risk of event between treatment and control group, which means that exp(δ_i_) will be the relative risk between treatment and control group. Furthermore, the δ_i_ are assumed to follow a normal distribution with mean δ and a random-effects standard deviation .

For treatment and control group we assume that the number of events of interest follow a binomial distribution given the true risk of event of the respective group and the number of patients. This means that we have the following model for analysis:

(1)


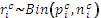

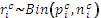

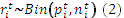

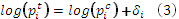


With r^c^_i_ and r^t^_i_ standing for the number of events in control and treatment group, p^c^_i_ and p^t^_i_ for risk of event in control and treatment group, and n^c^_i_ and n^t^_i_ for the number of patients in control and treatment group.

By defining the model on relative risk scale one needs to constrain δ_i_
so that p^t^_i_ is between 0% and 100%, that is in the interval [0,1]. This means that δ_i_ must be smaller than –log(p^c^_i_). This can be achieved replacing δ_i_  in equation (3) with min(δ_i_, –log(p^c^_i_)) to get:


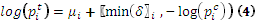


We implemented this model in a fully probabilistic (Bayesian) approach which will provide posterior probability distributions given the model and the data, but requires that prior distributions are defined for all parameters in the statistical model. For the control groups of each study, we set vague priors on the log of the odds of the event risk using normal distributions centred at zero with variance of 1000. A vague prior distribution was also used for the mean of the random-effects distribution by using a normal distribution centred at zero with variance 1000.

In many situations the available data makes it difficult to estimate the between study heterogeneity which is captured by the standard deviation . Therefore, the result for the posterior distribution of  is sensitive to the choice of the prior distribution for . Based on the discussion of others about the use of priors for , we conducted the analyses using three different weakly informative prior distributions for [^3^](#_ENREF_3)[^4^](#_ENREF_4) Recommendations exist to use plausible priors for  that put a small probability (e.g., 5%) on scenarios that are basically equivalent to assuming no relationship among the study specific parameters.[^5^](#_ENREF_5)[^6^](#_ENREF_6) Therefore, we used three different versions of weakly informative priors for , one with a mean of 0.5 and two with a mean of 1.

1. In version one, we used a half-normal distribution with a mean of 0.5 and a 95% interval from 0.02 to 1.4. We report the results of version one in the main body of text and tables
2. In version two, we used an exponential distribution for  with parameter 1, which has 2.5^th^ and 97.5% percentiles of 0.025 and 3.69
3. In version three, we used a uniform distribution between 0 and 2.

Results after using these different versions of weakly informative priors for  are shown in the table below. Results remain consistent.

|  Prior | Half-normal distribution | | Exponential distribution | | Uniform distribution between 0 and 2 | |
| --- | --- | --- | --- | --- | --- | --- |
|  | with mean of 0.5 | | with lamba of 1 | |  |  |
| **Outcome** | **RR (95% CrI)** | **^^ ** | **RR (95% CrI)** | **^^ ** | **RR (95% CrI)** | **^^ ** |
| Ischaemic Stroke, ICH or Death* | 0.38 (0.12 - 0.80) | 0.82 | 0.34 (0.09 - 0.62) | 0.22 | 0.31 (0.05 - 0.61) | 0.47 |
| Ischaemic Stroke | 0.49 (0.13 - 1.14) | 0.40 | 0.30 (0.07 - 0.78) | 0.15 | 0.28 (0.05 - 0.71) | 0.31 |
| ICH | 0.08 (0.00 - 0.45) | 0.22 | 0.00 (0.00 - 0.01) | 0.13 | 0.00 (0.00 - 0.02) | 1.11 |
| TIA | 0.86 (0.24 - 2.44) | 1.66 | 0.72 (0.04 - 3.19) | 2.23 | 0.71 (0.08 - 3.21) | 2.34 |
| Death | 0.73 (0.22 - 1.98) | 0.22 | 0.68 (0.01 - 2.00) | 0.43 | 0.57 (0.03 - 2.01) | 1.18 |
| Ischaemic Stroke or ICH | 0.24 (0.05 - 0.66) | 1.40 | 0.14 (0.00 - 0.38) | 0.49 | 0.12 (0.01 - 0.35) | 0.77 |
| Ischaemic Stroke or TIA | 0.64 (0.20 - 1.58) | 1.88 | 0.39 (0.04 - 1.34) | 2.07 | 0.36 (0.05 - 1.33) | 2.22 |
| Ischaemic Stroke, ICH or TIA | 0.45 (0.15 - 1.09) | 2.22 | 0.17 (0.01 - 0.75) | 2.44 | 0.17 (0.02 - 0.74) | 2.50 |

Here we show results when using an informative prior for the mean of the random effects distribution. As an informative prior we used on such that a prior distribution of the RR lies with 95% probability between 0.2 and 5 (with the half-normal distribution with mean 0.5 for the prior auf tau).

| Prior on mean of ln(RR) | Normal distribution with mean 0 and  standard deviation = √1000 = 31.62 | | Normal distribution with mean 0 and  standard deviation = 0.8211 | |
| --- | --- | --- | --- | --- |
| **Outcome** | **RR (95% CrI)** | **^^ ** | **RR (95% CrI)** | **^^ ** |
| Ischaemic Stroke, ICH or Death* | 0.32 (0.12 - 0.63) | 0.18 | 0.39 (0.20 - 0.70) | 0.12 |
| Ischaemic Stroke | 0.29 (0.08 - 0.77) | 0.13 | 0.41 (0.18 - 0.87) | 0.11 |
| ICH | 0.00 (0.00 - 0.05) | 0.18 | 0.24 (0.07 - 0.68) | 0.18 |
| TIA | 1.06 (0.33 - 2.92) | 1.62 | 1.05 (0.42 - 2.45) | 1.59 |
| Death | 0.69 (0.23 - 1.99) | 0.18 | 0.79 (0.33 - 1.91) | 0.16 |
| Ischaemic Stroke or ICH | 0.15 (0.04 - 0.41) | 0.22 | 0.26 (0.11 - 0.54) | 0.15 |
| Ischaemic Stroke or TIA | 0.59 (0.19 - 1.45) | 1.74 | 0.69 (0.29 - 1.47) | 1.64 |
| Ischaemic Stroke, ICH or TIA | 0.34 (0.10 - 0.88) | 2.03 | 0.47 (0.20 - 1.01) | 1.84 |

For the formal assessment of heterogeneity of treatment effect by study level characteristics we extended formula (4) which relates the risk in the control with the risk in the treatment group by adding a parameter β to model how the treatment effect varies by unit increase in the study level characteristic (studylevelchar) in the following way:^5^


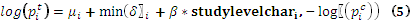


Again, a vague prior distribution was chosen for β, by using a normal distribution with mean 0 and variance 1000.

The variables used to describe study level characteristics were the following pre-specified dichotomous study characteristics (1=yes, 0=no): prospective design; enrolment of consecutive patients; inclusion of all enrolled patients in the analysis according to the principle of intention-to-treat; and balanced size of treatment groups.

Further we investigated heterogeneity of treatment effect for the primary composite outcome according to a post hoc definition of overall methodological quality, distinguishing between studies of high overall quality that satisfied all five criteria (prospective design, consecutive patients, intention-to-treat analysis, balanced group size and large sample size overall) and studies of low quality that did not.

Based on the posterior distribution for β, we obtained an estimate of the posterior probability that β is smaller (or larger) than zero. This tail probability we multiplied by 2 and used is as a Bayesian analogue of a p-value for effect modification also called p-value for interaction.

Moreover, we performed a “best evidence synthesis” by restricting the analysis of all outcomes to studies of high overall quality.^7^ Finally, we investigated whether there was evidence for confounding by indication (with patients with extensive strokes being potentially more likely to receive antiplatelets in view of their perceived high risk of intracranial hemorhage) by conducting separate analyses of overall mortality according to time-point of death (7 days or less versus 8 days or more after index event).

Between-trial heterogeneity was considered to be low if τ^2^ estimates were 0.04 or less, τ^2^ estimates of 0.14 may be interpreted as a moderate and 0.40 as a high degree of heterogeneity between studies. ^8-9^

Monte-Carlo Markov Chain simulation methods were used to obtain posterior distributions of the RRs for the outcomes of interest and for , τ^2^, and β (if β was included in the model).

We ran models with 3 chains. Results were obtained after a burn-in of 100 000 iterations, retaining every 10^th^ out of 500 000 iterations. Model convergence was assessed visually using the trace plots and using Gelman-Rubin plots. For all posterior distributions of parameters of interest we report the median, and the 2.5^th^ and the 97.5^th^ percentile of the posterior distribution.

Analyses were done using Stata version 11.0 and WinBUGS version 1.4.3.

Model code in WinBUGS

The code for the main analysis is as follows:

model{

for(i in 1:ns)

{

rc[i] ~ dbin(pc[i],nc[i])

rt[i] ~ dbin(pt[i],nt[i])

log(pt[i]) <- log(pc[i]) + min(delta[i],-log(pc[i]))

delta[i] ~ dnorm(d,precision.tau)

logit(pc[i]) <- mu[i]

mu[i] ~ dnorm(0,0.001)

}

tau2<-tau * tau

tau ~ dnorm(0,2.55 )I(0,)

precision.tau <- pow(tau,-2)

tauprior ~ dnorm(0,2.55)I(0,)

d ~ dnorm(0,0.001)

rr <- exp(d)

}

**References**

1. Lunn DJ, Thomas A, Best N, Spiegelhalter D. WinBUGS - A Bayesian modelling framework: Concepts, structure, and extensibility. *Stat Comput* 2000;10(4):325-37.

2. Lunn D, Spiegelhalter D, Thomas A, Best N. The BUGS project: Evolution, critique and future directions. *Stat Med* 2009;28(25):3049-67.

3. Lampert PC, Sutton AJ, Burton PR, Abrams KR, Jones DR. How vague is vague? A simulation study of the impact of the use of vague prior distributions in MCMC using WinBUGS. *Stat Med* 2005;24(15):2401-28.

4. Gelman A. Prior distributions for variance parameters in hierarchical models. *Bayesian Analysis* 2006;1(3):515-34.

5. Smith TC, Spiegelhalter DJ, Thomas A. Bayesian approaches to random-effects meta-analysis: a comparative study. *Stat Med* 1995;14(24):2685-99.

6. Neuenschwander B, Capkun-Niggli G, Branson M, Spiegelhalter DJ. Summarizing historical information on controls in clinical trials. *Clin Trials* 2010;7(1):5-18.

7. Slavin RE. Best-Evidence Synthesis: An Alternative to Meta-Analytic and Traditional Reviews. *Educational Researcher* 1986;15:5-11.

8. Spiegelhalter D, Abrams K, Myles J. *Bayesian approaches to clinical trials and healthcare evaluation*. Chichester: John Wiley & Sons, 2004.

9. Stettler C, Allemann S, Wandel S, Kastrati A, Morice MC, Schomig A, et al. Drug eluting and bare metal stents in people with and without diabetes: collaborative network meta-analysis. *BMJ* 2008;337:a1331.
