# Supplementary material for: Antiplatelets versus Anticoagulants for the Treatment of Cervical Artery Dissection: Bayesian Meta-Analysis
Source: PLoS One. 2013 Sep 5;8(9):e72697. doi: 10.1371/journal.pone.0072697 (PMC3764185; doi:10.1371/journal.pone.0072697)
Supplement: Appendix S4 — Different approaches for estimating summary effect. (DOC) [file pone.0072697.s004.doc]

S4. **Different approaches for estimating summary effect**

|  | Bayesian Random (main analysis presented) | | Fixed Effect Bayesian | Mantel Haenszel without continuity correction | Mantel Haenszel with continuity correction of sum 0.01 | Logistic Regression |
| --- | --- | --- | --- | --- | --- | --- |
| **Outcome** | **RR (95% CrI)** | **τ^2^ ** | **RR (95% CrI)** | **RR (95% CrI)** | **RR (95% CrI)** | **OR (95% CrI)** |
| Ischaemic Stroke, ICH or Death * | 0.32 (0.12 - 0.63) | 0.18 | 0.39 (0.20 - 0.66) | 0.40 (0.19 - 0.81) | 0.41 (0.21 - 0.83) | 0.38 (0.19 - 0.79) |
| Ischaemic Stroke | 0.29 (0.08 - 0.77) | 0.13 | 0.31 (0.11 - 0.74) | 0.33 (0.13 - 0.89) | 0.34 (0.13 - 0.90) | 0.32 (0.12 - 0.86) |
| ICH | 0.00 (0.00 - 0.05) | 0.18 | 0.00 (0.00 - 0.03) | N/A* | 0.06 (0.00 - 1.22) | 0.22 (0.00 - 1.37) |
| TIA | 1.06 (0.33 - 2.92) | 1.62 | 1.26 (0.72 - 2.36) | 1.45 (0.85 - 2.49) | 1.44 (0.85 - 2.44) | 1.70 (0.81 - 3.60) |
| Death | 0.69 (0.23 - 1.99) | 0.18 | 0.81 (0.31 - 2.03) | 0.96 (0.27 - 3.46) | 0.97 (0.27 - 3.46) | 0.97 (0.35 - 2.67) |
| Ischaemic Stroke or ICH | 0.15 (0.04 - 0.41) | 0.22 | 0.18 (0.06 - 0.42) | 0.23 (0.08 - 0.62) | 0.24 (0.09 - 0.63) | 0.20 (0.07 - 0.56) |
| Ischaemic Stroke or TIA | 0.59 (0.19 - 1.45) | 1.74 | 0.85 (0.51 - 1.36) | 0.93 (0.58 - 1.49) | 0.93 (0.59 - 1.49) | 0.91 (0.49 - 1.69) |
| Ischaemic Stroke, ICH or TIA | 0.34 (0.10 - 0.88) | 2.03 | 0.52 (0.31 - 0.83) | 0.72 (0.45 - 1.14) | 0.72 (0.46 - 1.14) | 0.61 (0.32 - 1.19) |

*Primary endpoint

N/A=could not be estimated

ICH=intracranial haemorrhage; TIA=transient ischaemic attack
